# Bladder metastasis without hematuria, following radiation therapy for lung adenocarcinoma

**DOI:** 10.1007/s13691-021-00490-2

**Published:** 2021-05-27

**Authors:** Takahiro Kirisawa, Manabu Okada, Chisako Miura, Ichiro Miura

**Affiliations:** 1 Department of Urology, Hokkaido Social Work Association Obihiro Hospital, 2 Higashi 5-jo Minami 9-chome, Obihiro-shi, Hokkaido, Japan; 2 Department of Pathology, Hokkaido Social Work Association Obihiro Hospital, 2 Higashi 5-jo Minami 9-chome, Obihiro-shi, Hokkaido, Japan

**Keywords:** Bladder metastasis, Hematuria, Lung adenocarcinoma, Pollakiuria, Secondary tumor

## Abstract

Bladder metastasis from lung adenocarcinoma is extremely rare. Unlike primary bladder urothelial tumors, the initial symptoms of this disease vary, and include pelvic pain, dysuria, and hematuria. There are few reports on cases without microscopic hematuria. An 86-year-old woman with a previous history of radiation therapy for lung adenocarcinoma complained of urinary frequency. A urinalysis was negative for hematuria and pyuria; thus, overactive bladder was suspected. However, the patient’s symptom worsened considerably, and cystoscopy revealed bladder tumor. Transurethral resection of the bladder tumor was performed. Based on the histological, immunohistochemical examination and clinical history, the final pathological diagnosis was bladder metastasis from lung adenocarcinoma. The patient died 19 days after the operation due to severe disease progression. In this rare case, a patient with bladder metastasis from lung adenocarcinoma did not show microscopic hematuria. Cystoscopy and computed tomography helped to make a rapid and accurate diagnosis.

## Introduction

Secondary cancers of the bladder are rare. The majority of cases occur due to direct spread from adjacent organs such as the prostate, lower gastrointestinal tract, and uterine cervical carcinomas. On the other hand, a smaller proportion of cancer metastasis occur due to lymphogenous or hematogenous pathways, as is sometimes observed in patients with skin, stomach, breast, or lung cancer. Secondary bladder neoplasms are reported to represent 2.3% of all malignant bladder tumors [1]. Cases of lung origin (squamous cell carcinoma, adenocarcinoma, small cell carcinoma) represent 2.8% of all secondary bladder neoplasms [1]. The most commonly reported symptom of bladder metastasis from lung cancer is gross or microscopic hematuria [2]. We herein report a case in which a patient was diagnosed with bladder metastasis from lung adenocarcinoma, without microscopic hematuria, in which the only symptom at presentation was pollakiuria.

## Case report

**Fig. 1:**
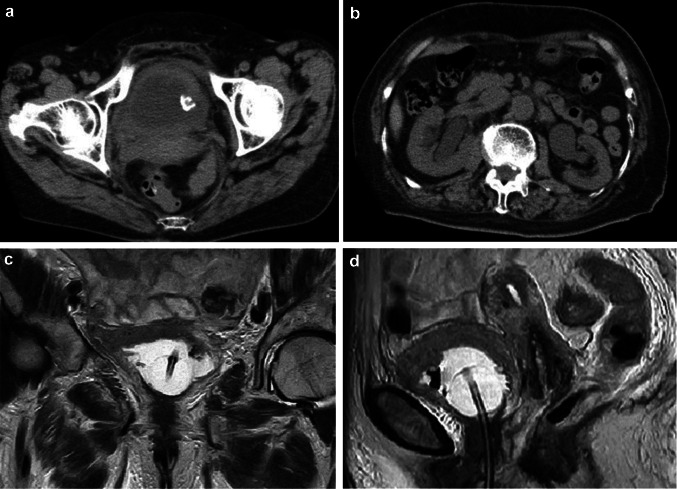
CT revealed 9 × 7 mm bladder tumor at the left top of the bladder and showing thickening of the whole bladder wall (**a**) and bilateral hydronephrosis (**b**). MRI showed the bladder trigone at coronal view (**c**) and relationship between bladder and uterus at sagittal view (**d**). These views ruled out locally advanced uterine cervical cancer

**Fig. 2:**
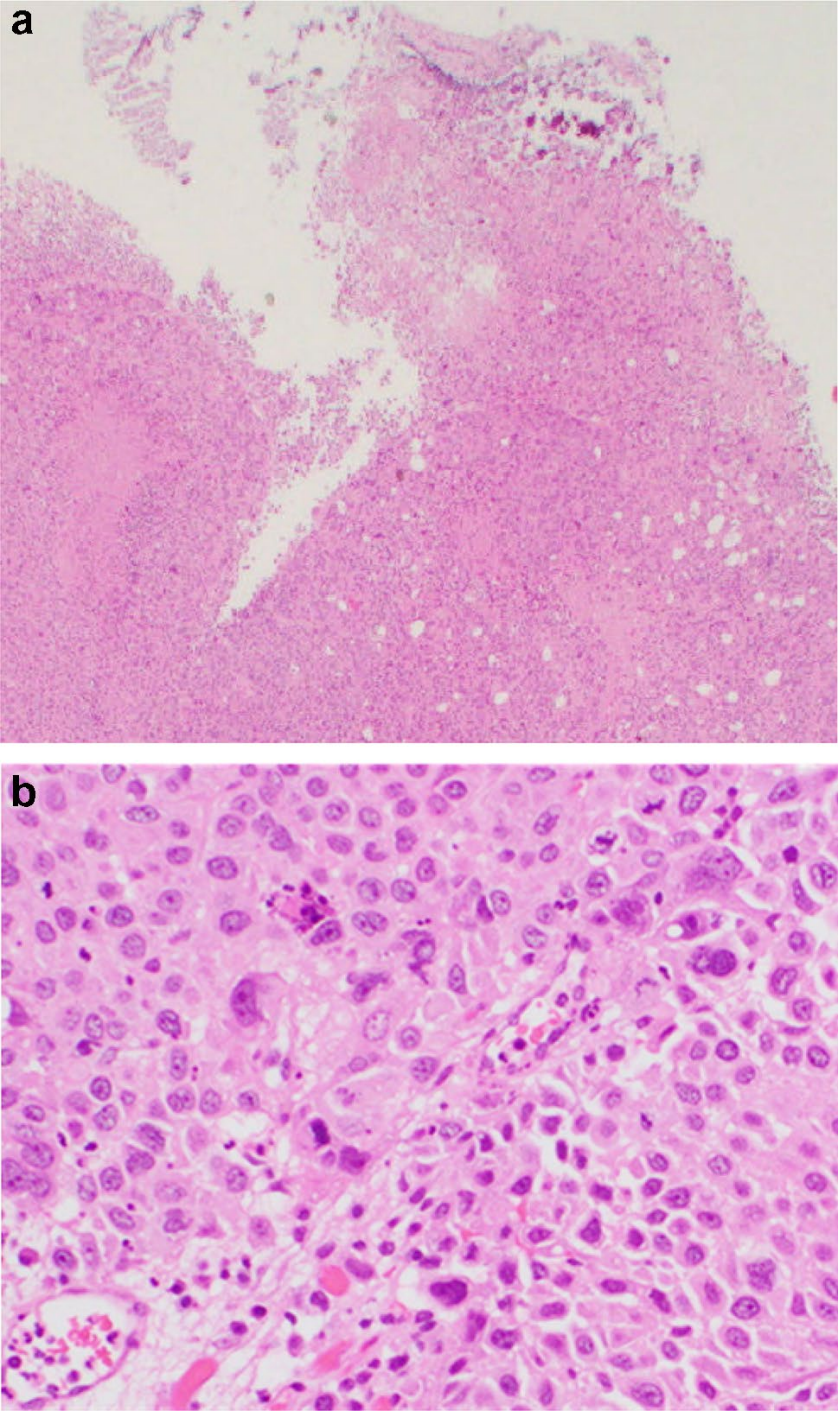
TURBT specimen. Tumor was mostly located in the lamina propria and muscularis propria. Ulcer was not detected at the superficial mucosa. Tumor shows nodular and sheet-like growth pattern with massive necrosis (**a**, HE staining, × 1.25), and large cells with pleomorphic enlarged nuclei with conspicuous mitosis (**b**, HE staining, × 40)

**Fig. 3:**
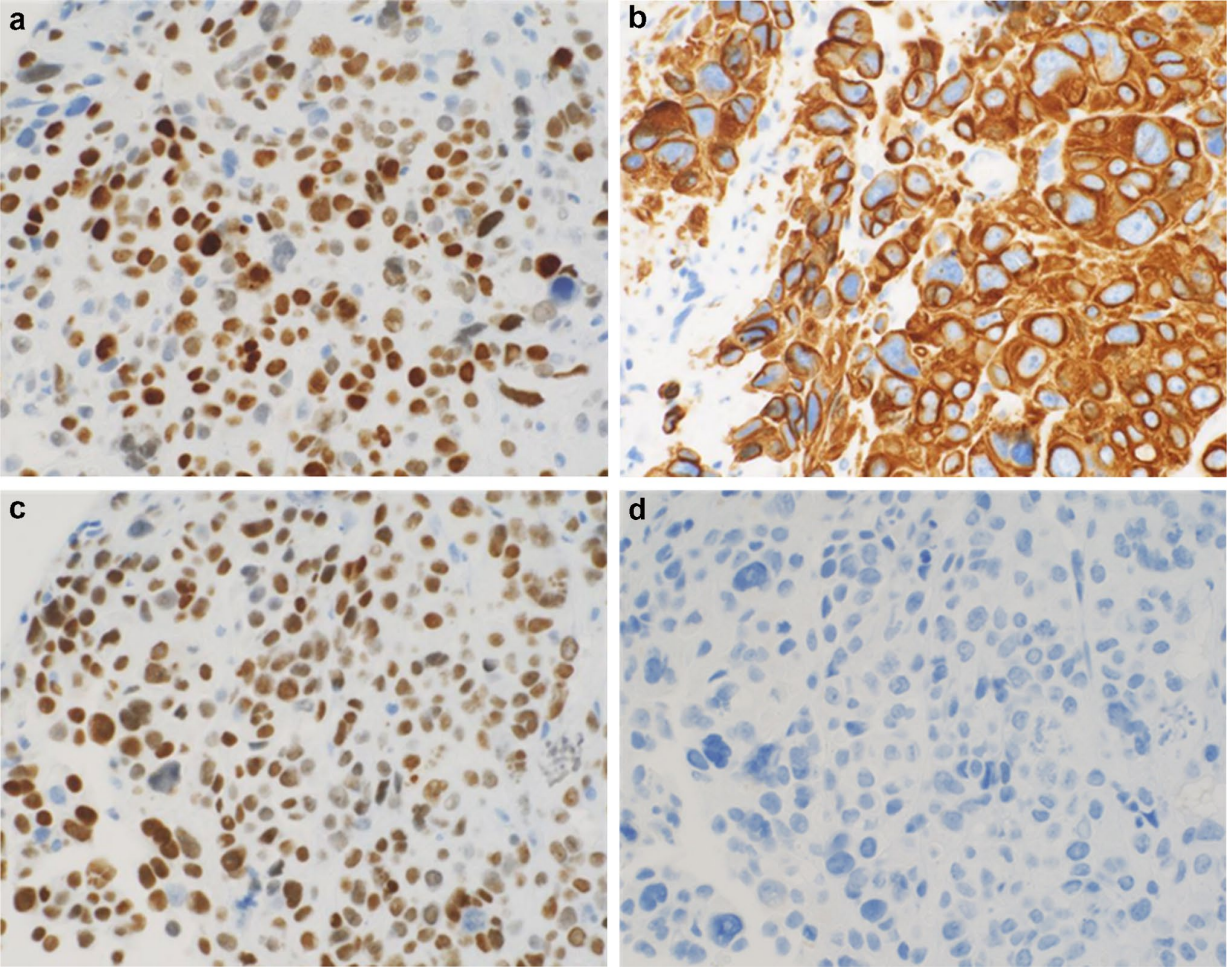
Immunohistochemical staining of tumor cells was positive for TTF-1 (**a**), CK7 (**b**), and GATA3 (**c**), while negative for CK20 (**d**)

An 86-year-old woman was diagnosed with left lung adenocarcinoma, stage T2aN0M0 in October 2018. One month after diagnosis, she received radiation therapy, without chemotherapy. In July 2020, she was referred to our department complaining of urinary frequency and urge incontinence, which had persisted for 1 week. A urinalysis was negative for hematuria and pyuria, therefore overactive bladder was suspected at first examination. Urine cytology was also negative. At return visit, investigations revealed normocytic anemia (hemoglobin 10.4 g/dl, mean cell volume 98.9 fl, white cell count 5.54 × 10^3^/μl, and platelet 203 × 10^3^/μl). Renal and liver function except for albumin were normal (creatinine 0.89 mg/dl, blood urea nitrogen 17.0 mg/dl, C reactive protein 0.39 mg/dl, aspartate aminotransferase 22 IU/l, alanine aminotransferase 14 IU/l, alkaline phosphatase 170 IU/l, and albumin 3.5 g/dl). Meanwhile, the value of lactate dehydrogenase was abnormal (251 IU/l). Frequency voiding chart revealed a total of 14 voiding episodes per day, with an average voided volume of 20 ml. Due to the severe and worsening symptoms, imaging studies were performed to detect organic disease. Cystoscopy showed a nodular tumor covered with calcification at the left top wall and mucosal folding change that involved the whole posterior wall. CT revealed a tumor (9 mm × 7 mm) at the dome of bladder, thickening of the bladder wall (Fig. 1a), and right hydronephrosis. Tumor marker levels were within the normal range (carcinoembryonic antigen 3.1 ng/ml, CA19-9 3U/ml, CA125 13U/ml, SCC 1.5 ng/ml, NSE 12.5 ng/ml), which was the same as that at diagnosis of lung carcinoma. 10 days later the patient was debilitated by bilateral back pain, fatigue, and anorexia. CT revealed bilateral hydronephrosis (Fig. 1b). RP showed extensive bilateral strictures at the lower part of the ureters. MRI showed distinct boundary between bladder and uterus (Fig. 2a, b), and Papanicolaou stain of uterine cervix cytology was also negative, then presence of locally invasive cervical cancer was denied. CT and MRI revealed no space occupying lesion except for the bladder. Double-J stents were inserted bilaterally and TURBT was performed. The bladder wall was considerably solid and maximum bladder capacity was approximately 20 ml. HE staining showed a nodular and sheet-like growth pattern with massive necrosis in mainly lamina propria. Tumor cells were large round cells with pleomorphic enlarged nuclei and conspicuous mitosis (Fig. 2a, b). The tumor invaded the muscle layer of the bladder wall. The findings were consistent with poorly differentiated carcinoma. An immunohistochemical examination of formalin fixed paraffin embedded sections revealed that the tumor cells were positive for thyroid transcription factor (TTF-1), cytokeratin 7 (CK7), and GATA binding protein-3 (GATA3), and negative for cytokeratin 20 (CK20) (Fig. 3a–d), Napsin A, and Uroplakin III.

**Fig. 4:**
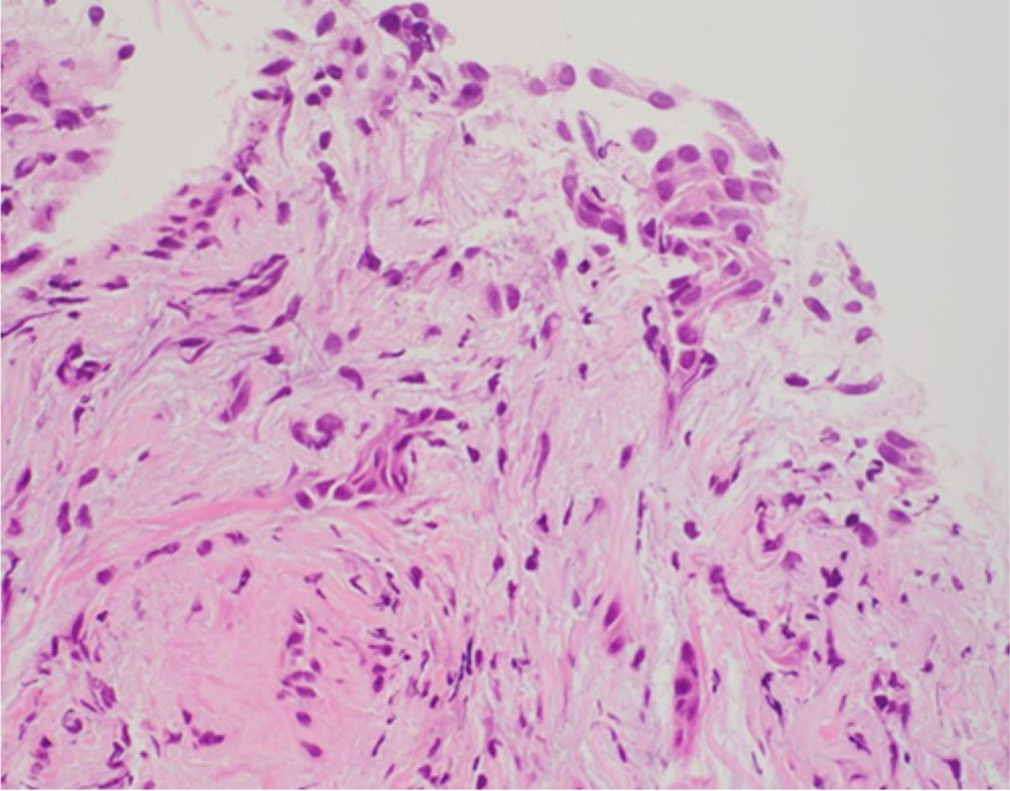
Lung biopsy specimen (HE stain × 40) show spindle cells infiltrating with active fibroblast proliferation, which was diagnosed lung adenocarcinoma

**Fig. 5:**
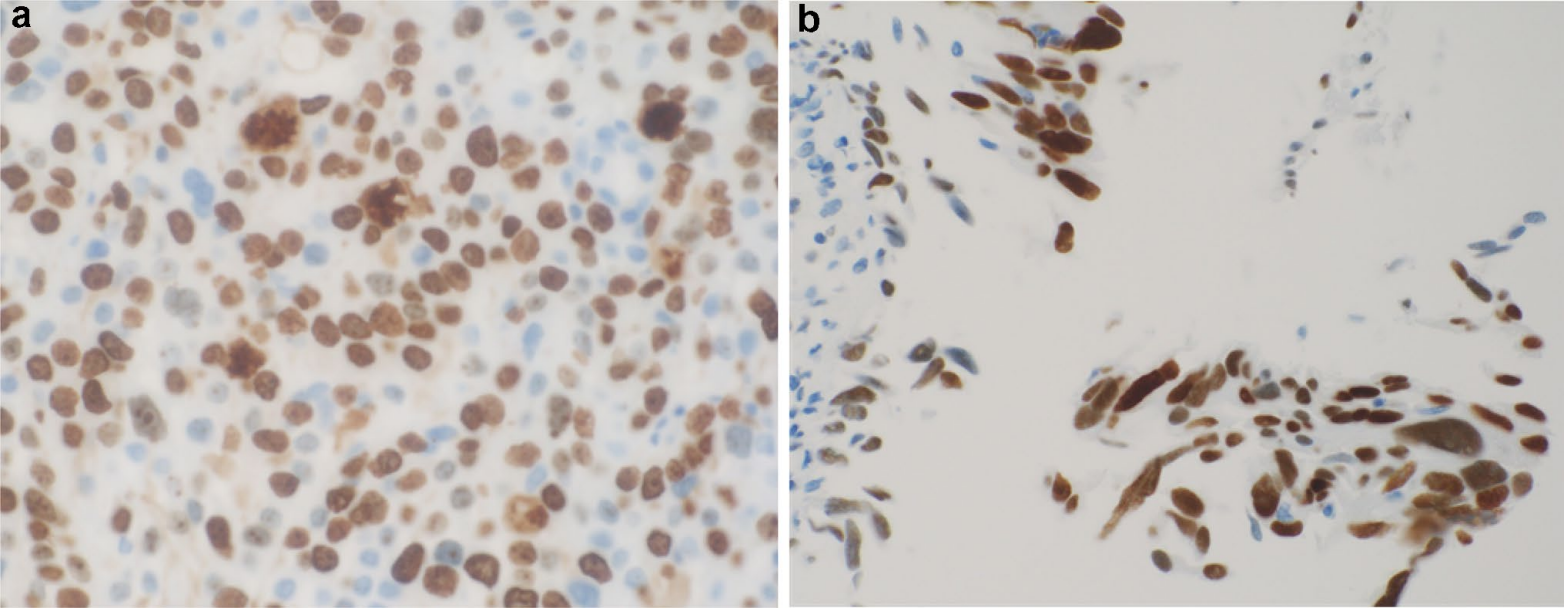
Immunohistochemical staining of tumor cells was positive for MIB-1 (**a**) and p53 (**b**)

As the primary lung cancer biopsy specimen was histologically high-grade malignancy findings with spindle-shaped cells as the main component and myxoid interstitium (Fig. 4), while cancer cells in metastatic lesion consist of large circular nuclei and eosinophilic cytoplasm, and MIB-1 index is 82% and p53 index is 83% (Fig. 5a, b). According to the results of study and clinical examination, the bladder tumor was diagnosed as metastasis of lung adenocarcinoma. The patient died 19 days after TURBT due to severe disease progression.

## Discussion

Gross hematuria is the most commonly reported presenting symptom in patients with bladder metastasis of lung adenocarcinoma [3, 4]; however, a few studies have reported cases without gross hematuria on presentation [5, 6]. In the present case, the patient presented with pollakiuria alone, without gross or microscopic hematuria. A possible reason for the absence of microscopic hematuria is that neoplastic cells coming through the hematogenous route are typically located in the lamina propria and/or muscularis propria of the bladder wall [2]. In the present case, the tumor grew widely in the lamina propria, and the superficial mucosa did not become an ulcer (Fig. 2a), so it is considered that there was no bleeding. The disease progression has been very rapid; thus, the tumor that is likely to have been localized to the lamina propria at the first visit invaded the bladder lining at the time of surgery. Aggressive imaging studies should therefore be considered for patients with a previous history of lung adenocarcinoma who present urinary or lower abdominal symptoms, even if a urinalysis reveals no microscopic hematuria. When cystoscopy fails to detect a tumor definitively, CT or MRI may be more sensitive [2].

Bladder cancer metastasis from lung cancer is difficult to distinguish from primary bladder adenocarcinoma. Primary bladder adenocarcinoma accounts for 0.5–2% of all primary bladder malignancies [7]. The histological features are not useful for differentiating between primary or secondary bladder tumors; thus, immunohistochemistry is crucial to establish a diagnosis. Several markers, used alone or in combination, are reported to be useful for the diagnosis of primary lung adenocarcinoma. TTF-1 is an important transcription factor in the early development of the fetal lung, and plays a crucial role in the molecular pathogenesis of the lung [8]. Moreover, Yue-Chiu Su et al. reported that a combination of TTF-1 positivity, CK7 positivity, and CK20 negativity was highly associated with primary pulmonary adenocarcinoma [9]. In their study, the sensitivity and specificity of this combination of antibodies for a primary lung adenocarcinoma were 60% and 100%, respectively. Another study also reported a similar result in relation to this immunohistochemistry pattern [10]. This pattern was the same as the present case.

Meanwhile, relationship between patient’s catastrophic disease progression and tumor malignant grade is an issue to be considered. An association between the expression of GATA3 and the survival rate in primary lung cancer patients was reported by Hashiguchi et al. [11]. Their study demonstrated that the high expression of GATA3 and vascular invasion were independently associated with poor OS. In the present case, more than 50% of the tumor cells were positive for GATA3 and vascular invasion was also confirmed, which are consistent with the poor prognosis reported by Hashiguchi et al. In addition, the high index of MIB-1 and p53 staining in the present bladder specimen suggests that the metastatic lesion was more malignant. These immunohistochemistry were likely to reflect aggressive invasive behavior of this neoplasm.

In this rare case, a patient with bladder metastasis from lung adenocarcinoma presented with pollakiuria, without microscopic hematuria. When a patient with a previous history of lung adenocarcinoma presents any urinary or lower abdominal symptoms, secondary bladder tumor should be considered in the differential diagnosis, even if urinalysis is negative for microscopic hematuria.

## Data Availability

Records and data pertaining to this case are in the patient’s secure medical records in Obihiro Kyokai Hospital.

## Abbreviations

CT: Computed tomography
MRI: Magnetic resonance imaging
OS: Overall survival
RP: Retrograde pyelography
TURBT: Transurethral resection of the bladder tumor
